# Observed Impact of Long-Term Consumption of Oral Cannabidiol on Liver Function in Healthy Adults

**DOI:** 10.1089/can.2021.0114

**Published:** 2023-02-06

**Authors:** Robert Kaufmann, Keith Aqua, Jeff Lombardo, Martin Lee

**Affiliations:** ^1^ Midwest Allergy Sinus Asthma PC, Springfield, Illinois, USA.; ^2^ Valid Care LLC, Denver, Colorado, USA.; ^3^ Syzygy Research Solutions, LLC, Wellington, Florida, USA.; ^4^ University of Buffalo, Buffalo, New York, USA.; ^5^ UCLA Fielding School of Public Health, Los Angeles, California, USA.

**Keywords:** ALT, AST, CBD, cannabidiol, liver function test, LT

## Abstract

**Introduction:**

Previous studies have suggested that prescribed cannabidiol (CBD) products may cause elevations in liver tests (LT). This study compared the prevalence of elevated LT in an adult population self-administering CBD with the normal and general adult population prevalences.

**Materials and Methods:**

Adults 18–75 years of age across the United States taking CBD orally for a minimum of 30 days were recruited from 12 individual CBD product companies in this decentralized, observational study and sent their standard CBD regimen from the company of their choice. An app-based, 21CFR Part 11 decentralized clinical study platform (ValidCare Study) was used to securely automate consent inclusion/exclusion criteria and collect all the data for this study, including: demographic information, medical history, reasons for taking, dosage, current medications dosage, adverse effects, and efficacy. At the end of 30 days, LTs were obtained. Follow-up LTs were offered to all individuals with elevated alanine transaminase (ALT) values.

**Results:**

A total of 28,121 individuals were invited to participate in this study, 1475 enrolled, and 839 (female: 65.3%, male: 34.7%) completed the study. Full-spectrum hemp oil was used by 55.7%, CBD-isolate by 40.5%, and broad spectrum by 3.8%. The mean±SD daily dose of CBD was 50.3+40.7 mg. The prevalence of elevated ALT was 9.1%, aspartate aminotransferase (AST) 4.0%, alkaline phosphatase 1.9%, total bilirubin 1.7%, with 85.5% of the ALT elevations <2×the upper limit of normal (ULN) with only 0.3% having ALT levels >3× ULN. The prevalence of ALT and AST elevations (9.1% and 4.0%) were not significantly different from known adult general population prevalences (8.9% and 4.9%). There was no significant association between CBD dosage and LT values. Thirty-three individuals with elevated ALT levels had follow-up LT performed with 21 having normal LT, 8 having the same severity of ALT elevation, and 4 having an increase in severity, 1 of which ultimately became normal.

**Conclusions:**

Self-medication of CBD does not appear to be associated with an increased prevalence of LT elevation and most of the LT elevations are likely due to the conditions/medications for which the individuals are taking CBD.

## Introduction

Cannabidiol (CBD) self-medication is becoming increasingly common in the United States with surveys showing that the percent of individuals who have tried CBD has increased from 14% in 2019 to at least 33.3% in 2020.^1,2^ Although a very-high-dose CBD is associated with elevations in liver tests (LTs) in children being treated for epilepsy and in normal adults, all other studies of CBD use have found no such association.^3,4^ With the sole exception of diarrhea, all of the adverse outcomes in childhood epilepsy studies (abnormal liver function tests, somnolence, sedation, and pneumonia) were limited to instances where CBD may have interacted with other medications, such as clobazam and/or sodium valproate.^3^

In a phase 1 trial of 70 normal adults who consumed 1500 mg/day of CBD along with various epileptic drugs, not a single individual developed abnormal LT, however, in a similar phase 1 trial of adults consuming only CBD at the same dose/day, 7 of 16 individuals (44%) had elevated LT with 5 having levels meeting criteria for drug-induced liver injury.^4,5^ Why such vast differences in findings exist is unknown at this time, however, these very high daily doses of CBD are much greater than the daily dosage typically consumed by CBD self-medicating users in the United States.

Laboratories set the 97.5% value of LT in adult individuals with no disease as the upper limit of normal (ULN). However, the prevalence of elevated LT in the general adult populations is estimated at between 10% and 20% and has been rising over the years.^6,7^ The prevalence of elevated alanine aminotransferase (ALT) and elevated aspartate aminotransferase (AST) in the United States in years 1999–2002 were 8.9% and 4.9%, respectively.^7^ Using any LT by itself as a screening instrument for liver disease or damage is not as useful as using multiple LTs, that is, ALT, AST, alkaline phosphatase (ALP), and total bilirubin (TB).^3^ This study was undertaken to determine the prevalence of elevated levels of these four LTs in an adult population of self-medicating CBD users.

## Materials and Methods

Adults, 18–75 years of age across the United States, known to be taking CBD orally for a minimum of 30 days, were recruited from consumers of 12 individual CBD product companies to participate in this decentralized, observational, IRB-approved study in accordance with the ethical standards on human experimentation. An app-based, 21CFR Part 11 decentralized clinical study platform (ValidCare Study) was used to securely automate consent inclusion/exclusion criteria.

Exclusion criteria included individuals with known liver disease, liver function impairment, allergies to CBD, or taking the following: valproate, Vitamin A, clobazam, cyclosporin, phenytoin, fluvoxamine, isoniazid, ritonavir, clarithromycin, diltiazem, erythromycin, grapefruit juice, itraconazole, ketoconazole, nefazodone, ritonavir, telithromycin, or verapamil. Individuals selected were sent their standard CBD regimen (full-spectrum, broad spectrum, or CBD-isolate) from the company of their choice. Demographic information, medical history, reasons for taking, dosage, and current medications were collected through the app, along with daily journaling information on dosage, adverse effects, and efficacy.

At the end of 30 days of journaling, blood draws were performed locally and serum was sent to one of two national laboratories, where LTs were performed. The different laboratories had different ULN values for their populations. To normalize the ALT data from the different laboratories, ALT values were adjusted to the percentile of the ULN for their laboratory. All individuals with elevated ALT values were contacted and offered a follow-up LT. Medical history, medication history, and cannabinoid use history data were collected for the time period between the testing points. Individuals were encouraged to provide information on LT ordered by personal medical providers, regardless of whether they consented to the follow-up testing.

Quantitative data were analyzed using the Wilcoxon rank-sum or signed-rank tests and qualitative data by the chi-square test for homogeneity or a binomial test using an exact *p*-value calculation. Nonlaboratory variables analyzed include weight, height, body mass index (BMI), sex, age, form of CBD, composition of CBD, alcoholic drinks per day, number of medical conditions, number of prescribed drugs, number of other therapies, number of over-the-counter treatments, and number of other CBD treatments being used.

Different laboratories had different ULN values for their populations. To normalize the ALT data from the different laboratories, ALT values were adjusted to the percentile of the ULN for their laboratory. A forward stepwise linear regression was used to identify predictors of LT values. The algorithm was validated by repeated application to subsamples.

## Results

**FIG. 1.**
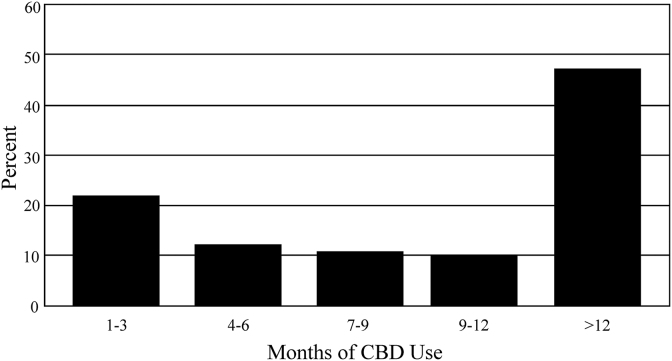
Percent of individuals versus the length of their CBD use.

A total of 28,121 individuals from across the United States were invited to participate in this study. One thousand four hundred seventy-five were enrolled, and 839 fully completed the study with blood draws for LTs. The 839 individuals who completed the study came from 43 states, including Alaska and the District of Columbia. Of these, 548 (65.3%) were female and 291 (34.7%) were male. Age ranged from 18 to 75 with the mean±SD of 45.5+13.1 years. There was no statistical difference in age between females and males (45.8±13.2 and 44.8±12.7, respectively). The percent of individuals versus the length of their CBD use is shown in Figure 1.

Table 1 shows the number and percent of individuals taking the various compositions of CBD and compares the average daily doses. Full-spectrum hemp oil was taken by 55.7%, CBD-isolate by 40.5%, and broad-spectrum CBD by 3.8%. Overall, the mean±SD daily dose of CBD (mg) was 50.3+40.7 mg/day. Full-spectrum users' daily dose was 40.0+36.8 mg, CBD-isolate users (63.9+42.8 mg,), and broad-spectrum users (56.5+26.5 mg). The upper limit of the range for each group is several times that of the mean.

**Table 1.** Number of Individuals and Average Daily CBD Dose by Composition of CBD

Composition of CBD	*N*	%	CBD dosage (mg/day)				*p* vs. FS
			Mean	SD	Range	Median	
All	839	100%	50.3	40.77	2.5–390	36.3	NA
FS	467	55.7%	40.0	36.82	2.5–210	40.1	NA
CBD isolate	340	40.5%	63.9	42.89	15–390	50.0	<0.001
Broad spectrum	32	3.8%	56.5	26.50	16.5–140	50.0	<0.001

CBD, cannabidiol; FS, full spectrum; SD, standard deviation.

The forms by which the different compositions of CBD were taken are listed in Table 2. Almost half (49.7%) of the participants used a tincture, whereas 22.1% used a capsule or pill, 13.9 used an edible formulation, 12.6% used a nanotechnology-treated product, and 1.7% used an additive that could be added to a slushy or food.

**Table 2.** Composition and Average Daily Dose for Different Forms of CBD

Form	Participants		Composition			Average daily dose (mg)		
	*n*	%	Full spectrum	Broad spectrum	CBD isolate	Mean	s.d.	Range
Tincture	417	49.7	273	18	126	54.0	38.3	8–245
Capsule/pill	185	22.1	88	0	97	65.4	49.2	24–390
Edible	117	13.9	0	0	117	49.8	25.5	23–135
Nano	106	12.6	106	0	0	7.6	3.0	2.5–25.5
Additive	14	1.7	0	14	0	70.0	28.0	50–140

The average daily dose of the nanotechnology-treated CBD was significantly lower than any of the other forms of CBD used. This nanotechnology-treated CBD was a full-spectrum product. When it was removed from the analysis, the average daily dose of full-spectrum CBD increased to 63.2+41.8 mg. There was no statistical difference in average dosage between the different compositions or forms of CBD used when the nanotechnology-treated CBD was excluded from the analysis.

Table 3 shows the prevalence of the LT in this study. The number and percentage of individuals with elevated LT were: ALT 9.1%, AST 4.0%, ALP 1.8%, and TB 1.4%. The prevalences of elevated ALT and AST were significantly higher than the 2.5% prevalence in a normal population with no medical conditions (*p*<0.00001 for ALT and 0.005 for AST). However, they were not significantly different from their reported prevalences in the general adult U.S. population (8.9% for ALT and 4.9% for AST).^6^

**Table 3.** Prevalence of Elevated LFT as Compared to Normal and General Populations

LFT	n	%	Normal population, %	*p*	General adult population, %	*p*
ALT	76	9.1	2.5	<0.001	8.9	0.605
AST	34	4.0	2.5	0.005	4.9	0.227
ALP	15	1.8	2.5	0.194		
TB	12	1.4	2.5	0.041		
ALT or AST	84	10.2			9.8	0.697

ALP, alkaline phosphatase; ALT, alanine transaminase; AST, aspartate aminotransferase; LFT, liver function test; TB, total bilirubin.

The prevalence of TB (1.4%) was significantly less that the normal population prevalence of 2.5% (*p*<0.05), and the prevalence of ALP was not different than the normal population prevalence of 2.5%. The prevalence of those having either an elevated ALT or AST was 10.2% (86 individuals), which was not statistically different than the reported prevalence in the general adult U.S. population (9.8%).^6^

Table 4 shows the prevalence of abnormal LT for different levels of elevation of ALT. Of 76 individuals with elevated ALT, 65 were <2× ULN, 8 were between 2×and 3× ULN, and 3 individuals were >3× ULN. The percentage of individuals having an elevated ALT with an elevated AST decreased as the level of ALT decreased. In the group of individuals with ALT >3× ULN, 100% also had an elevated AST; this percentage was decreased to 81.8% for those with ALT >2× ULN and was only 25.0% for those with ALT >1× ULN.

**Table 4.** Prevalence of Elevated LT for Different Levels of ALT Elevation

ALT >1× ULN			
Laboratory test	No. of subjects	% of total population	% of those with ALT >1× ULN
Elevated ALT	76	9.06%	100.0%
Elevated AST	24	2.86%	30.4%
Elevated ALP	3	0.36%	4.0%
Elevated TB	1	0.12%	1.3%

ALT >2× ULN			
Laboratory test	No. of subjects	% of total population	% of those with ALT >2× ULN
Elevated ALT	11	1.31%	100.0%
Elevated AST	9	1.07%	81.8%
Elevated ALP	0	0.00%	0.0%
Elevated TB	0	0.00%	0.0%

ALT >3× ULN			
Laboratory test	No. of subjects	% of total population	% of those with ALT >3× ULN
Elevated ALT	3	0.36%	100.0%
Elevated AST	3	0.36%	100.0%
Elevated ALP	0	0.00%	0.0%
Elevated TB	0	0.00%	0.0%

ULN, upper limit of normal.

The only elevations of either TB or ALP were found in those with an ALT >1× ULN. The one TB elevation in the patients with elevated ALT was very minor being only 0.1 mg/dL higher than the ULN of <1.2 mg/dL. No one in either the >3× ULN or >2× ULN groups had an elevated ALP.

**FIG. 2.**
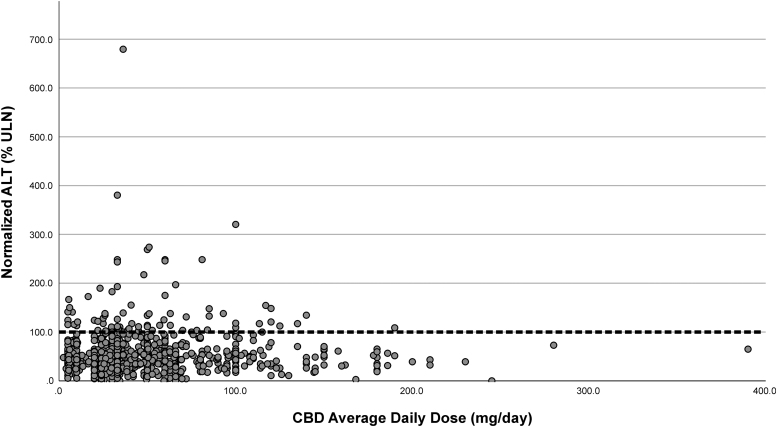
A scatter plot of the average daily dose (mg/day) and the normalized ALT level (%ULN). The dotted line represents the ULN for ALT. ALT, alanine transaminase; ULN, upper limit of normal.

Although BMI, age, and gender were highly correlated with ALT percentile level, multiple regression adjustment found that only BMI (*p*<0.001) and age (*p*<0.05) had an effect on predicting elevated ALT percentile levels. As is illustrated in Figure 2, there was no statistical correlation between the percentile level of ALT and the daily dose of CBD. In addition, there was no significant correlation with any of the other values, including length of use and percentile ALT level.

The mean value for daily dose of individuals with elevated AST was 52.3+41.0 mg/day and 50.1+36.3 for those with normal AST. There was no significant difference in the prevalence of elevated LT between companies (Table 5), nor was there any difference between CBD compositions (Table 6). There were, however, significant correlations between ALT levels and AST and ALP levels but not with TB. All the LT values were reasonably approximated by a normal (Gaussian) distribution, but the ALT distribution had an extended right tail, which corresponds to the increased incidence of elevated ALT levels as compared with a normal, healthy population.

**Table 5.** Prevalence of Elevated LT by Brand of CBD

Brand	No. of participants	No. with ALT > ULN	Prevalence
1	43	5	11.60%
2	55	5	9.10%
3	106	9	8.50%
4	84	5	6.00%
5	63	4	6.30%
6	145	17	11.70%
7	69	5	7.20%
8	70	7	10.00%
9	15	2	13.30%
10	53	4	7.50%
11	54	7	13.00%
12	82	6	8.40%
Total	839	76	9.10%

**Table 6.** Prevalence of Elevated LT Level by CBD Composition

CBD composition	>1× ULN		>2× ULN		>3× ULN	
	n	Prevalence	n	Prevalence	n	Prevalence
Full spectrum	39	8.4%	6	1.28%	2	0.43%
Broad spectrum	2	6.3%	1	3.13%	0	0.00%
CBD-isolate	35	10.3%	4	1.10%	1	0.29%
Total	76	9.1%	11	1.31%	3	0.36%

It should be noted that one individual had an LT drawn at week 2 of the study for reasons that are unknown and reported that their LTs were abnormally high. However, he continued in the study and at week 4 when his LTs were drawn for this study, his LTs had returned to normal.

The number (percentage) of individuals with medical conditions (as per their history) was 585 (69.7%) of the total subjects and the average number of medical conditions per person was 2.7+2.34. The number (percentage) of subjects with medical conditions with normal ALT values was 539 (70.6%) with a mean of 2.7+2.3, while those with elevated ALT values was 46 (60.5%) and 2.8+2.72, respectively.

Similarly, the number and percentage of subjects taking prescription drugs was 525 (62.6%) overall, 476 (62.4%) for individuals with normal ALT levels, and 49 (64.5%) for those with elevated ALT levels. Overall, an average of 2.4+2.3 drugs per person was taken, while those with normal ALT levels took 2.4+2.3 drugs and those with elevated ALT levels took 2.3+1.8 drugs. There were no significant differences between any of these values.

Of the 76 individuals with elevated LTs, 33 agreed to have follow-up LTs performed by our laboratories (27) or by their local physician (6). The remaining 43 individuals either refused to agree to have a follow-up LT or never showed up at the laboratory for the test.

Of the individuals having follow-up LT data, one individual had an ALT 3× ULN, an elevated AST, and normal ALP and bilirubin levels on the first set of LT, stopped CBD for 4 months and continues to have essentially the same levels of LT. Two individuals had an initial ALT >2× ULN and <3× ULN and both had normal LT on follow-up. One continued on CBD and the other, who was also consuming large amounts of alcohol throughout the study, stopped taking CBD and reduced her alcohol intake.

All the remaining individuals (30) had initial ALT levels >1× ULN and <2× ULN and none of these individuals had stopped CBD because of their ALT levels. Of these, seven continued to have an elevated ALT >1× ULN and <2× ULN. One individual's ALT increased to >2× ULN and <3× ULN. This individual had an elevated LT before starting the study due to acetaminophen toxicity and restarted the acetaminophen between the first and the second LT.

Three other individuals also admitted to having had elevated LT in the past, but claimed they had not indicated such when being screened for the study because they knew it would exclude them from participating. Three individuals' repeat ALT increased to >3× ULN, two of whom started consuming marijuana products between the two tests and the other had a third set of LT which was normal even though she was continuing on her CBD. This last individual was taking three drugs (lisinopril, meloxicam, and glimepiride), which are known to be associated with elevated LTs.

There was no relationship between continuing to take CBD, daily dose of CBD and ALT levels or change in ALT elevation severity. There were no differences in any of the initial data in the severity of LT elevation between those that had follow-up LT performed and those that did not. Therefore, ultimately 30 of the 33 individuals (91%) had their ALT levels ultimately return to normal or remain minimally elevated (<2× ULN). The three individuals with significantly elevated ALT (>3× ULN) had reasons to explain their continued elevation that were not related to CBD consumption, as described above.

Of the 1475 individuals enrolled in the study, 33 (2.2%) reported an adverse reaction, of which 31 were classified as unrelated to CBD ingestion with two being classified as possibly related. These two consisted of one case of atrial fibrillation and one case of constipation and psychoactive effects.

## Discussion

Although it would have been ideal to have a controlled, double-blinded clinical trial to study this issue, at the time of design and beginning performance of this study, CBD was considered a class 5 drug in the United States. Therefore, even though CBD use was widespread, such a trial was difficult, if not impossible to perform using commercial CBD. Therefore, this observational study was thus performed in its stead.

The ALT data in this population study are similar to other population studies, which found that the level of ALT is affected by BMI, age, and gender.^8,9^ These studies also found significant correlations among ALT, AST, and ALP levels, which also were found in this study. These similarities suggest that the sample in this study is representative of the general adult population.

In this self-selected sample of individuals who were self-dosing CBD, there was an increased prevalence of LT elevation as compared with a normal healthy population with no medical conditions. However, individuals in this study were taking CBD primarily for medical reasons, making laboratory comparisons of this population to that of a normal healthy population unrealistic, as a large proportion of the individuals with medical conditions in the United States will have abnormal values.^10^ When compared with the general adult population norms in the United States, the prevalence of elevated ALT and/or AST was no different.

Although comparing the prevalence of elevated ALT to the general population seems illogical when the exclusion criteria for this study included “having a history of elevated LT,” such a comparison was made because several individuals knowingly falsified their history of elevated LT so that they could be included in the study. The transient nature of elevated LT in most individuals in this study is similar to that seen and reported in the general population and, in the majority, their LT reverted to normal even though CBD ingestion was continued.^6,7^ In the few individuals with persistent severely elevated or worsening severity of ALT elevations, the cause can easily be attributed to factors other than CBD.

In this study, the vast majority of participants with elevated ALT and/or AST had levels <2×UNL and among the few individuals with levels >2×UNL, none had any elevation of ALP or TB. There was no association between dose or length of CBD usage and ALT levels. Even though a number of individuals consumed large amount of CBD, there was no increase in prevalence of elevated AST levels. In fact, the daily mean dose and standard deviation were essentially the same in both those with normal and elevated AST levels.

CBD was not found to be a factor in determining ALT levels, not a single individual in this study had liver disease, and the prevalences of ALP and TB in this population of CBD users were lower than those found in the normal healthy population. This suggests that self-medication of CBD in some individuals may help prevent liver disease, as has been suggested in animal and *in vitro* studies.^11,12^ This paradoxical in effect on drug-induced liver damage with difference in dosing levels of CBD is not new.^13^

Many of the individuals in this study had been taking multiple drugs, including many that are known to cause LT elevations, and this fact has been a common theme in CBD-associated LT elevations in most studies when this association has been examined.^3,4^ However in these studies, the daily dose (10–20 mg/kg) was a significant factor in the association. In this study, the average daily dose was 0.65±0.57 mg/kg/day, which is an order of magnitude less and was comparable to other studies that examined the daily dose typically consumed by a self-dosing CBD user.

Although, it may be possible that CBD at lower doses can cause transient elevations in LT, the findings of this study support that they are more likely due to demographic, physical, and medical conditions already suffered by the individuals for which they are self-medicating with CBD for relief of associated symptoms. In addition, only 0.14% (2 of 1475) individuals had an adverse reaction that was considered to possibly have any causal relationship to taking CBD.

## Conclusions

Individuals self-medicating with CBD for various conditions use daily doses that do not appear to significantly increase the prevalence of elevated LT rather, the underlying conditions and/or other medications being taken for these conditions may be the cause for these abnormalities. Self-medication of CBD in a population of individuals at doses consumed in this study was not associated with clinical liver disease.

## Abbreviations Used

ALP: alkaline phosphatase
ALT: alanine transaminase
AST: aspartate aminotransferase
BMI: body mass index
CBD: cannabidiol
LFT: liver function test
LT: liver tests
TB: total bilirubin
ULN: upper limit of normal
1× ULN: one times the upper limit of normal
2× ULN: two times the upper limit of normal
3× ULN: three times the upper limit of normal
